# The immunobiology of humanized Anti-IL6 receptor antibody: From basic research to breakthrough medicine

**DOI:** 10.1016/j.jtauto.2019.100030

**Published:** 2019-12-23

**Authors:** Yoshiyuki Ohsugi

**Affiliations:** Ohsugi BioPharma Consulting Co., Ltd., 5th Fl. Denbo Bldg., 1-39-11 Asakusa, Taito-ku, Tokyo, 111-0032, Japan

## Abstract

The clinical use of monoclonal antibodies is well established in human medicine and has been amongst the most important contributions of basic science to clinical disease. One such antibody, the humanized anti-human IL-6 receptor antibody, is used to treat a variety of autoimmune diseases, particularly rheumatoid arthritis. It is extremely difficult and a laborious process to go from a concept at the research bench, to government approval. Such approval implies not only efficacy but, more importantly, an appropriate safety profile. In this review, the history of anti-human IL-6 receptor antibody is discussed in depth beginning with the author’s experience during a sabbatical visit at the University of California at Davis in 1978. At that time, it was discovered that B cell activation was at least one critical factor in the development of autoimmunity. Approximately six years later, the cDNA encoding for IL-6 was cloned as BSF-2 (B cell stimulatory factor 2) to differentiate B cells to produce antibody. Soon after, it was suggested that this cytokine plays an important role in the development of autoimmune diseases. Based on this evidence, the journey began to search for an IL-6 inhibitor. Although there were numerous obstacles in finding lead compounds, ultimately, basic science developed the methodology for high throughput readouts that would inhibit the biologic function of IL-6. It was finally concluded that a mouse monoclonal antibody against IL-6 receptor would be optimal. In 1991, this antibody was humanized by using CDR-grafting technology in collaboration with the MRC (Medical Research Council). The drug was named tocilizumab and launched as an innovative anti-rheumatic drug in 2008 in Japan. Subsequently, the drug has been used throughout the world and has achieved enormous success in helping patients who suffer from inflammatory arthropathies. The lessons learned in the development of this antibody have application to the study of biologics and their application to other human diseases.

## Highlights

• 30 years history of the research and development of humanized anti-interleukin-6 receptor antibody (tocilizumab).
• Breakthrough drug for the treatment of rheumatoid arthritis, vasculitis, Castleman’s disease and others.
• Developed in Japan and spread over world-wide including US and Europe.
• Commercial name: Actemra, and Ro-actemra in Europe.

## 1 Introduction

**Fig. 1:**
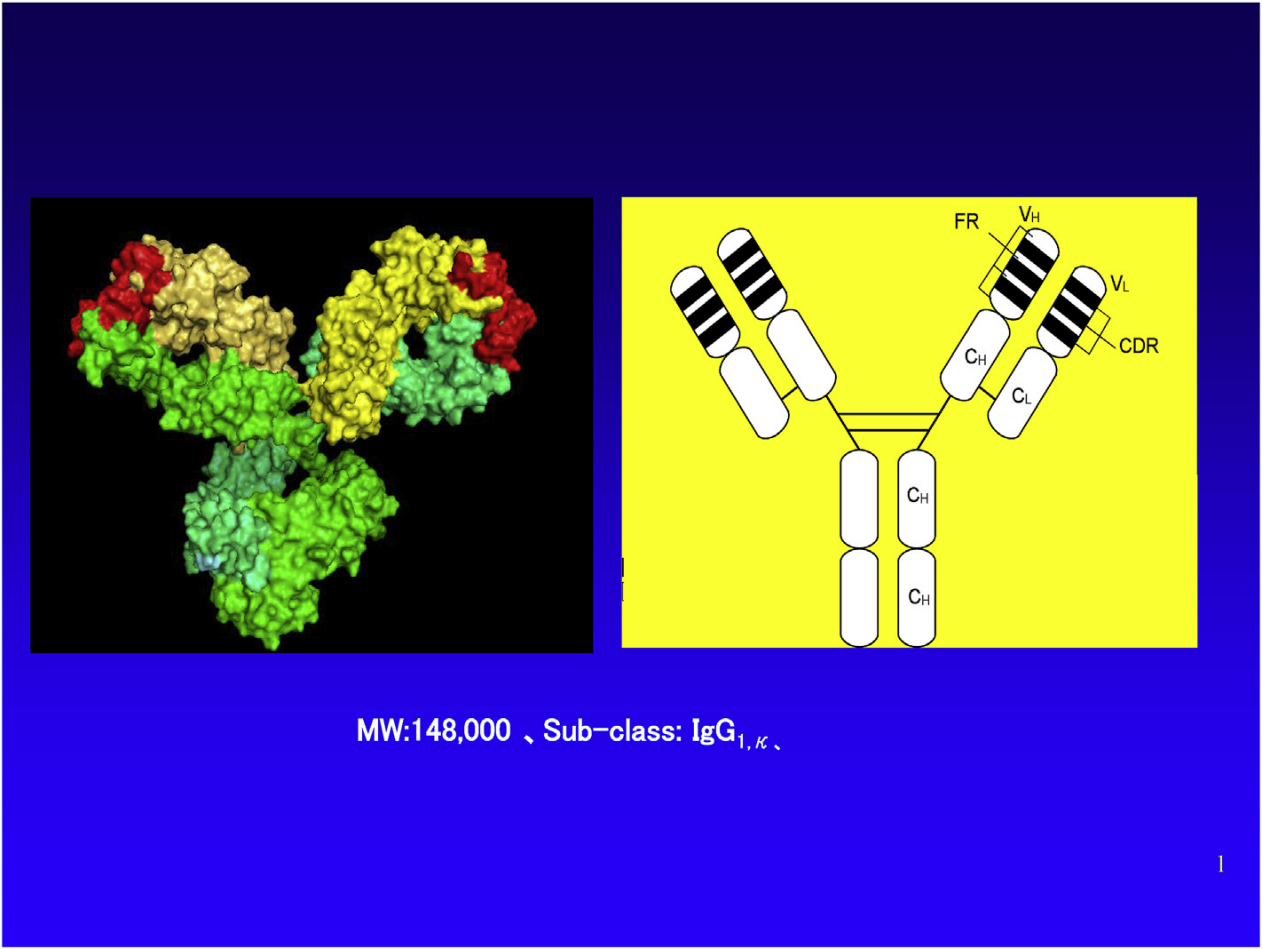
Molecular structure of tocilizumab. Right panel, schematic representation of the two-dimensional structure. Left panel, three-dimensional structural molecular model. (Produced by Drs. Ohta and Kobayashi, Chugai Pharmaceutical Co., Ltd.).

**Fig. 2:**
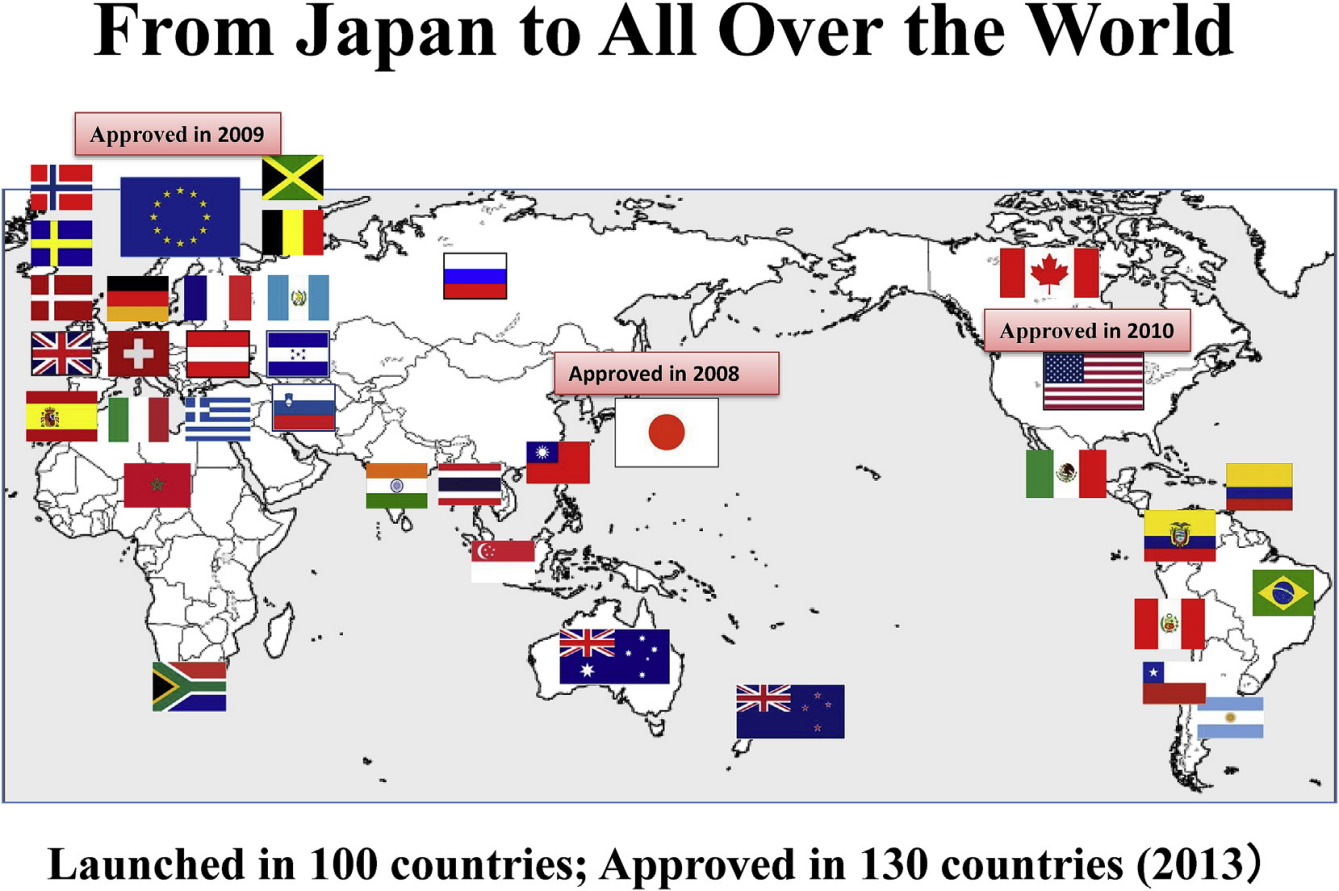
Tocilizumab approval/launch status: From Japan to all over the world Tocilizumab (trade name Actemra) was approved for RA in 2008 in Japan, which was the first approval in the world. EMEA approved it in 2009 (under the trade name of RoActemra) and FDA (Actemra) did it in 2010.

**Fig. 3:**
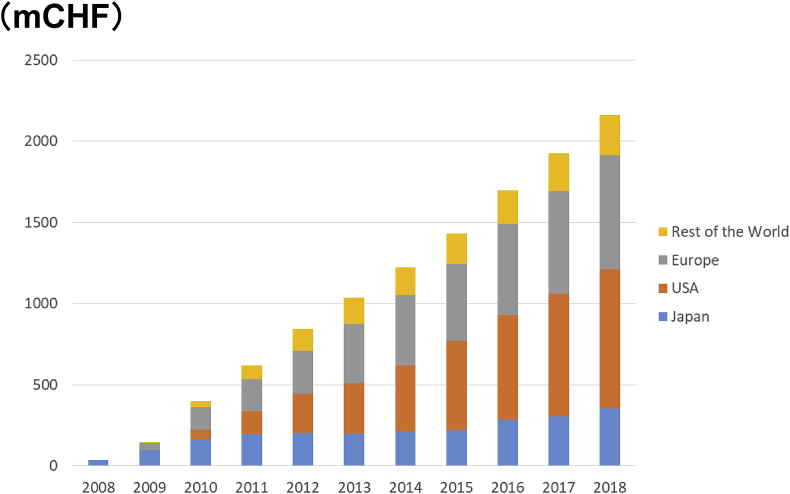
Sales of Tocilizumab. Currently, Actemra has been spreading all over the world (more than 100 countries). Around 700,000 patients with rheumatoid arthritis are receiving Actemra therapy.

Tocilizumab (trade name Actemra, and Ro-Actemra in Europe) is a drug discovered and developed by Japanese pharmaceutical company Chugai Pharmaceutical Co., Ltd. for the treatment of rheumatoid arthritis, polyarticular juvenile idiopathic arthritis, systemic juvenile idiopathic arthritis, Takayasu arteritis, giant cell arteritis, adult Still’s disease, inhibitor of CAR-T cell-induced cytokine release syndrome, and multicentric Castleman’s disease [1,2]. It is a humanized antibody against the human IL-6 receptor, manufactured by culturing Chinese hamster ovary (CHO) cells as genetic recombinants (Fig. 1). It inhibits the biological function of IL-6 by inhibiting the binding of IL-6 to the IL-6 receptor. It is the first-in-Japan biologic (a therapeutic antibody) and also had been until quite recently the only IL-6 inhibitor worldwide (see Fig. 2, Fig. 3).

The success rate of new drug development is extremely low, perhaps 1 in 30,000. It is particularly difficult to succeed in discovery of a revolutionary new drug to treat autoimmune diseases such as rheumatoid arthritis, because the etiology is not fully understood. It was my experience during my study abroad at UC Davis between 1978 and 1981 that triggered research into inventing inhibitors of the polyclonal B cell activation as a way to control autoimmunity. Tocilizumab, born in Japan and spread worldwide, has been launched in more than 90 countries and has been employed in the treatment of 650,000 patients with rheumatoid arthritis. It took 30 years to obtain FDA approval in 2010. In those 30 years, multiple other cytokines have been identified to play a role in autoimmune [3,4], autoinflammatory [5,6] and allergic diseases [7], as well as various cancers [8], and many biologics have been developed to target these cytokines [9,10]. Herein is the 30-year history of the development of tocilizumab from the start of the research to its commercialization.

## 2 History of cytokines

The first published lymphocyte-derived mediator was blastogenic factor (BF, later named IL-2), found in mixed leucocyte culture in 1965 [11]. Interferon-gamma was also identified as an interferon-like virus inhibitor the same year [12]. Macrophage migration inhibitory factor (MIF) was identified simultaneously in 1966 by John David and Barry Bloom [13,14]. In 1969, Dudley Dumonde proposed the term “lymphokine” as a name for lymphocyte-derived secreted proteins, and later macrophages and monocytes-derived proteins were called “monokines” [15]. In 1974, Stanley Cohen reported that MIF was produced in virus-infected allantoic membranes and kidney cells, indicating that its production was not limited to leukocytes. This led to a change of both of lymphokine and monokine to “cytokines” [15,16]. Later, some cytokines were named interleukins (IL), because of their origin from leukocytes and their action on leukocytes. Subsequently, they were numbered in order to their discovery date. The first two were IL-1, a macrophage activating factor and IL-2, a T-cell growth factor. We are now up to at least 35 interleukins and there are several other families of cytokines, such as the interferons mentioned above, and some growth factors, that are grouped according to their structure or function. Although IL-6 is a proinflammatory cytokine, it has many other functions as will be presented below.

## 3 The discovery of IL-6

The cDNA coding for IL-6 was performed by Hirano et al. of Osaka University in 1986 as BSF2 (B cell stimulatory factor 2), a factor that induces differentiation of B cells to produce antibody [17]. Later it was named IL-6 because it was the 6th interleukin discovered. Kishimoto was studying in the laboratory of Kimishige Ishizaka of Johns Hopkins University as a postdoctoral fellow, when Ishizaka discovered IgE, the antibody that induces allergy, in 1970. Kishimoto was engaged in research to clarify the mechanism of antibody production in rabbits. He later published a paper indicating that T cell-derived soluble factor acts on B cells to induce their proliferation and differentiation into antibody producing cells [18].

Kishimoto returned to Osaka University (Third Department of Internal Medicine) in 1975. Kazuyuki Yoshizaki, who had been studying human lymphocytes in the same department, utilized human B cells to clarify the mechanism of antibody production in humans. Thence, Yoshizaki used chronic lymphocytic leukemia (CLL) cells, rather than healthy B cells to construct a more sophisticated assay system. Leukemic cancer cells are monoclonal and express a uniform antibody with identical idiotypes on the cell surface. Therefore, if the cells are stimulated by anti-immunoglobulin (Ig) antibodies, all of the cells will be activated. Subsequently, Yoshizaki began to look for a T cell derived factor with the ability to stimulate B cells. He attempted to fractionate the supernatant of the T cell culture stimulated by phytohemagglutinin (T cell blastogenic factor).

In 1982, Yoshizaki et al. published a paper indicating that B cells, previously activated by anti-Ig antibody, proliferate and differentiate into antibody producing cells by adding T cell-derived soluble factor. Interestingly, in this study, it became obvious that the factor that proliferates B cells (BCGF) and the factor that promotes the differentiation of B cells and their conversion to antibody producing cells (BCDF) are different molecules. B cells stimulated with only BCGF did not become antibody-producing cells; antibodies were produced only when BCDF was added after B cells proliferation was induced by BCGF [19]. The discovery of BCDF was epoch-making, because at that time it was thought that once the B cell divides, it automatically differentiates into antibody-producing cells.

Hirano, at the same time, independently discovered B cell differentiation inducing factors in T-lymphocytes in the pleural effusion of tuberculosis pleurisy patients. In 1982, Hirano successfully isolated the TRF-like agent/BCDF [20], and joined Kishimoto’s laboratory. In April of the same year, Tetsuya Taga joined Kishimoto’s laboratory as a postgraduate student with an interest in BCDF. Taga was involved in the purification of BCDF using the cell lines CESS and SKW6-CL4. Taga established a new method of measuring IgG produced by CESS cells, an enzyme linked immunosorbent assay, or ELISA, in 1983 [21]. This method contributed to the success in the purification of BCDF. In 1984, they finally succeeded in obtaining a tiny amount of BCDF and determined the sequence of the N-terminal partial peptide. Finally, the cDNA for IL-6 was cloned [17]. Research on IL-6 was in an extremely severe competitive environment but it is noteworthy that Japanese researchers were leading this international competition.

## 4 The discovery of the IL-6 receptor and the signaling pathway of IL-6

One year later the receptor gene was identified [22] and based on these results, extracellular domains of the receptor of various lengths (soluble receptor) were prepared, and their inhibitory effects on IL-6 were examined. However, even by binding to and capturing IL-6, the function of IL-6 was not inhibited, but instead, induced transmission of IL-6 signals into the cell [23].

**Fig. 4:**
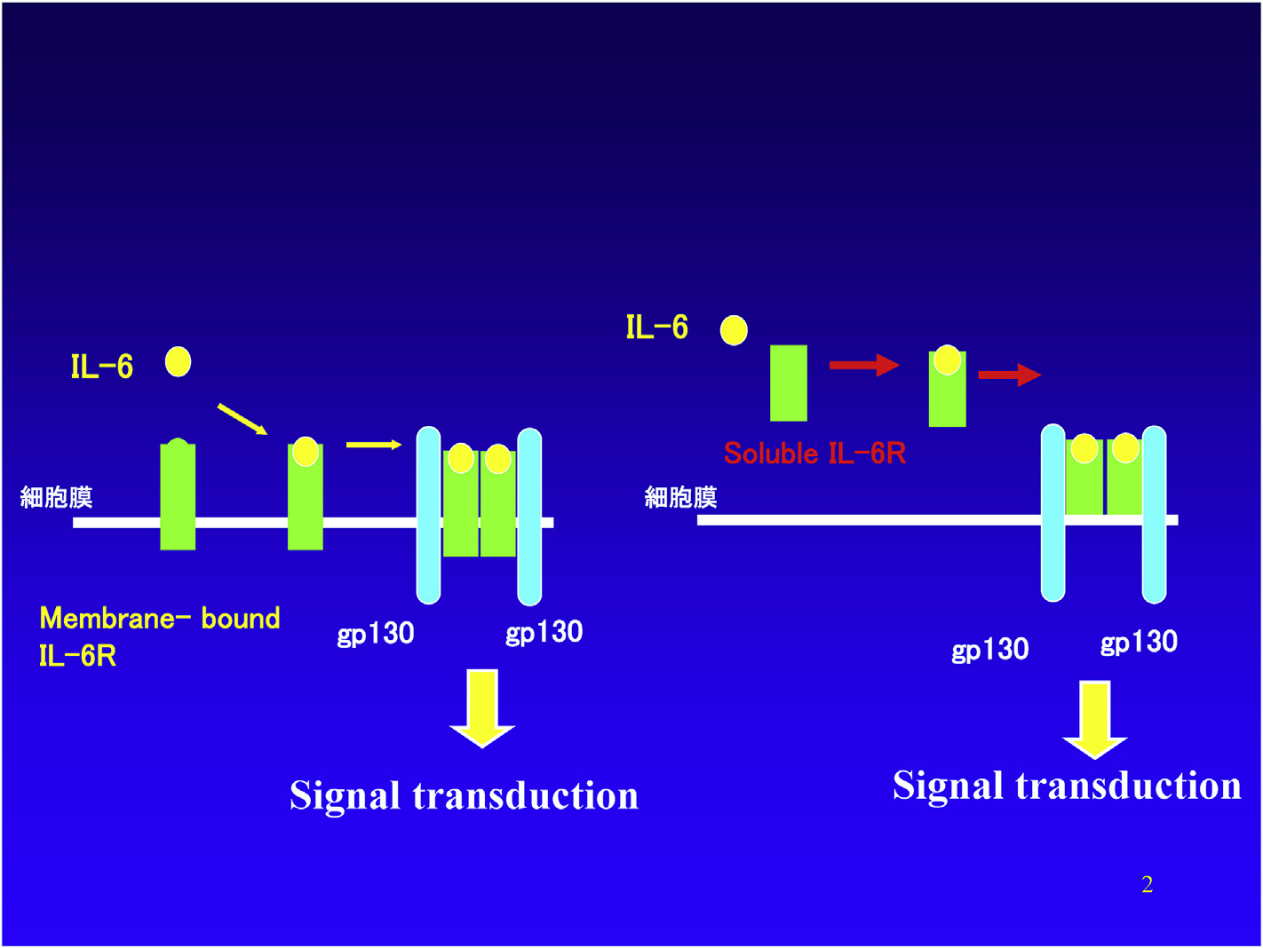
Pathway of intracellular signaling through IL-6 receptors. IL-6 binds to membrane-bound IL-6R and then associates with a secondary receptor known as gp130. When two of the resulting complexes combine to form a hexamer, the two gp130 molecules form a homodimer in the cytoplasmic region, the signal transmission system is activated, and the IL-6 signal is transmitted to the nucleus. Alternatively, soluble IL-6 receptor present in body fluids forms a complex with IL-6, and when that complex associates with gp130 on the cell surface, the IL-6 signal is transmitted into the cell.

The signal transduction pathway for IL-6 is unique. As shown in the left side of Fig. 4, for IL-6 signal transduction, two different kinds of membranous proteins are necessary [[24], [25], [26], [27]]. The first is the IL-6 receptor with molecular weight of 80-kDa (IL-6R, IL-6Rα-chain, CD126), which is able to bind to IL-6. The other is gp130 with molecular weight of 130-kDa (IL-6R β-chain, CD130) which is unable to bind IL-6 but is responsible for signal transduction. When IL-6 binds to membranous IL-6R (mIL-6R), that complex becomes able to bind to GP130 to form a trimer complex of IL-6, IL-6R, and gp130. Then, two of this trimer complex bind each other to form a hexamer complex. As a result, the intracellular domains of two gp130 molecules approach each other to make a homodimer inside the cells. Subsequently, the activation of enzymes that phosphorylates proteins, called JAKs, begin signal transmission within the cell. As a result, STAT-3, a transcription factor, is phosphorylated and activated STAT3 (pSTAT3) moves to the nucleus to transmit the IL-6 signal.

As noted on the right side of Fig. 4, a soluble IL-6 receptor (called a “soluble receptor” in contrast to the membrane-bound type receptor) can also bind to IL-6. This complex has the ability to associate with the gpl30 on the cell surface and transmits the IL-6 signal into the cell in the same manner as mIL-6 receptor does. Therefore, IL-6 exerts its effects through gpl30 even in cells that do not express IL-6 receptors on their cell surface. Soluble IL-6 receptor is able to transmit the signal in the same way as a membrane-bound receptor by binding with IL-6, which is why it does not work as an IL-6 inhibitor [[24], [25], [26], [27]].

In 2005, a crystal structure study clarified the 3-D formation of this hexamer complex [28]. Importantly, the soluble IL-6 receptor (sIL-6R), which lacks transmembrane and cytoplasmic domains, is present in serum and body fluids. Once sIL-6R binds to IL-6, the complex becomes capable of associating with gp130 to transduce the IL-6 signal into cells. This means that the gp130-mediated IL-6 signaling pathway works even for cells that do not express IL-6R on their cell surface. This is called trans-signaling [29].

It should also be noted that gp130 is expressed ubiquitously in all tissues, even in cells that lack detectable expression of IL-6R [27]. This suggests that gp130 is not merely a component of IL-6R and that it might function as a common signal transducer for various cytokines. In fact, several different cytokines share gp130. It has been reported that ciliary neurotropic factor (CNTF), leukemia inhibitory factor (LIF), oncostatin M (OM), IL-11, and cardiotropin-1 (CT-1) all use gp130 as a component of their receptors [18]. This explains why these cytokines have very similar activities (redundancy) [30].

## 5 Biology of IL-6: Involvement of IL-6 in the pathogenesis of autoimmune diseases in experimental animals

As mentioned above, IL-6 cDNA was originally cloned as a terminal B cell differentiation factor into antibody-producing plasma cells. This revealed that BSF-2/IL-6 was identical to hybridoma/plasmacytoma growth factor and hepatocyte-stimulating factor that had been independently studied under different names [1,2]. These subsequent studies reflected that IL-6 is produced not only by T cells, but also by other various types of cells including B cells, monocytes, macrophages, dendritic cells, fibroblasts, endothelial cells, glial cells, and several types of tumor cells. IL-6 also acts on a variety of cell types and regulates a wide range of biological functions, including immune-inflammatory response, hematopoiesis, and nervous system responses, as described below. This also suggested that IL-6 would have the ability to act on a variety of cells and mediate many different biological functions [[29], [30], [31]].

For example, IL-6-stimulated hepatocytes induce acute-phase reactants, including C-reactive protein, serum amyloid A protein, and fibrinogen, and decrease serum albumin levels. In addition, this cytokine has been found to enhance the synthesis of a peptide called hepcidin in the liver which regulates iron recycling in the spleen as well as absorption of iron from the intestine, resulting in iron deficiency anemia. It has also been shown that IL-6, in the presence of soluble IL-6 receptor, act on synovial fibroblasts to induce RANKL and secrete VEGF, resulting in osteoclast differentiation and neovasculization, respectively. In addition, a series of studies in mice demonstrated that IL-6 promotes the development of a subset of T-helper cells called Th17 ​cells that impact the pathogenesis of autoimmune diseases [1,2].

Animal studies have demonstrated that IL-6 is also critical in the development of experimental arthritis [[32], [33], [34], [35], [36], [37], [38], [39]]. The development of collagen-induced arthritis (CIA) is strongly inhibited in IL-6 deficient mice [32] and in mice treated with anti-mouse IL-6R antibody (MR16-1) [39]. IL-6 blockade also inhibits the development of antigen-induced arthritis [36], and the spontaneous development of autoimmune arthritis in SKG mice [37]. Furthermore, Atsumi and Hirano et al. found that continuous IL-6 signaling through GP130 spontaneously causes arthritis in mice [33].

It should be noted that treatment with rat monoclonal anti-mouse IL-6 receptor antibody (MR16-1) almost completely inhibited the appearance of anti-DNA autoantibody, resulting in the prevention of the development of lupus nephritis in NZB/NZW F1 mice [40].

## 6 The history of polyclonal B cell activation and its importance in autoimmunity

**Fig. 5:**
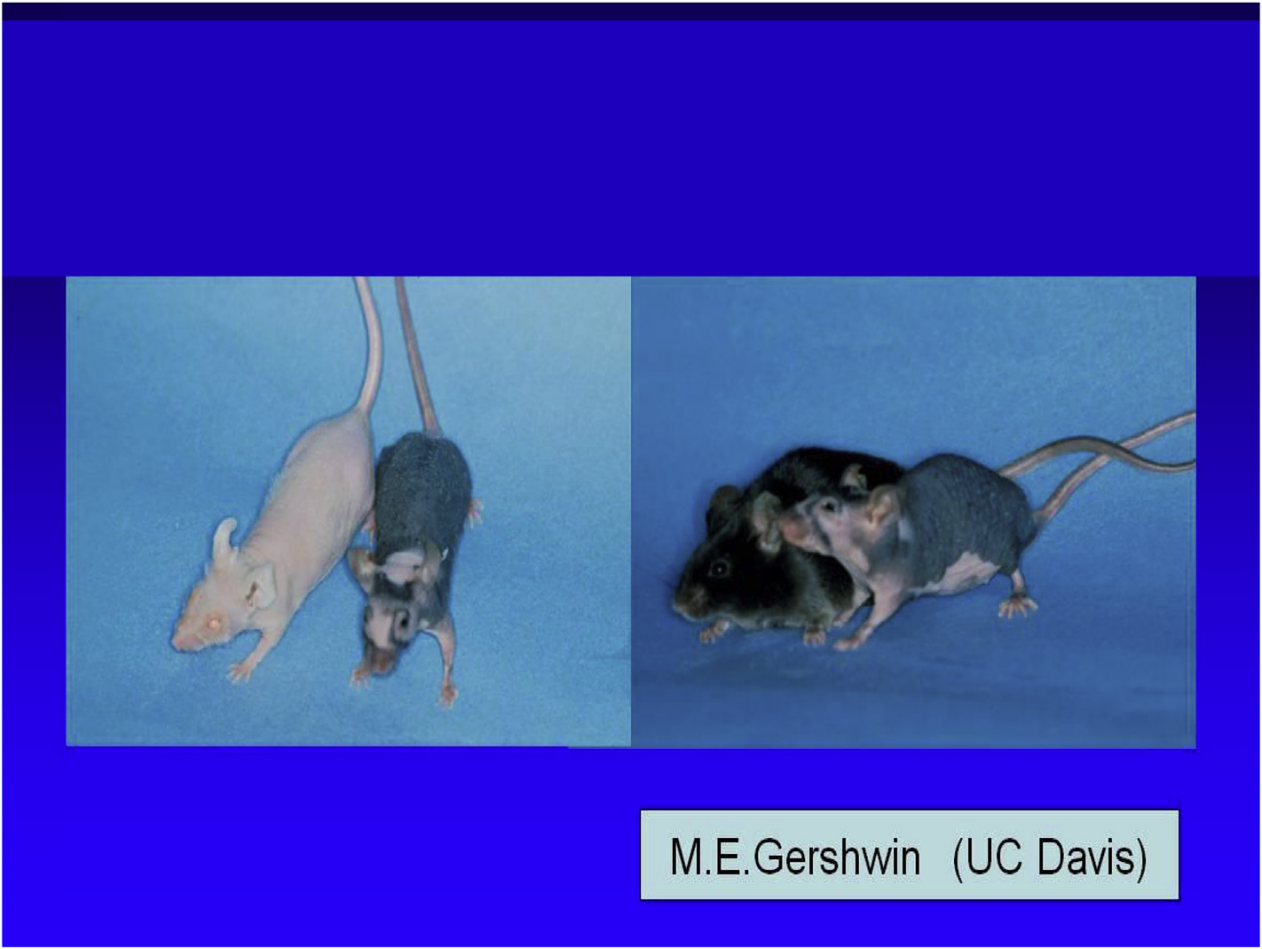
New Zealand mice transfected with nude gene.

**Fig. 6:**
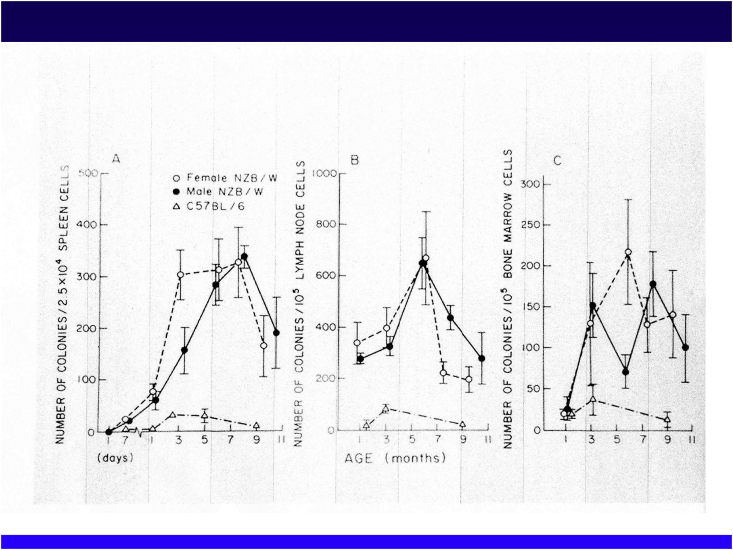
Primary B cell defect in NZB/NZWF1 mice. Experimental data showing increased numbers of B-cell colonies in NZB/NZWF1 mice from early life (left to spleens, nodes, and bone marrow). (J. Immunology, 1979; International Conference of immunology in Paris).

New Zealand mice and, in particular, NZB/NZWF1 mice, have been frequently used as models of systemic lupus erythematosus (SLE) (Fig. 5). Ohsugi and Gershwin of UC Davis investigated the B-cell function of these mice using colony formation methods [41]. NZB/NZWF1 mice form a greater number of B-cell colonies than control mice. B-cell abnormalities were observed in the spleen, lymph nodes and the bone marrow (Fig. 6). This anomaly was considered to be an intrinsic anomaly because it was recognized at the embryonic stage. Germ-free and athymic (nude) mice had similar B-cell activation abnormalities, suggesting that T cells and the microbiome were not involved [41]. This was an epoch-making discovery because the prevailing theory was that a lack of suppressor T cells was a cause of the autoimmunity.

In NZB/NZWF1 mice, anti-DNA antibodies appear at 4–5 months of age. This autoantibody forms immune complexes with DNA and attaches to the capillaries of the glomeruli of the kidney. At this point, complement is activated, and neutrophil infiltration triggers an inflammatory response that eventually progresses from glomerulonephritis to renal failure with death at 9 months.

Interestingly, New Zealand nude mice also developed nephritis and died of renal failure. The production of anti-DNA antibodies was also found to be comparable to that of wild-type mice, indicating that T cells are not essential for the production of autoantibodies [42]. This was the first experimental proof worldwide of polyclonal B cell activation in the absence of T cells and was published in 1980 as one of the major topics at the International Congress of Immunology in Paris. To confirm the data, NZB/NZWF1 mice transduced with the Xid (X-linked immunodeficiency) gene were established. Those defective in B cells produce immunoglobulin (Ig) M but are unable to produce IgG antibodies. Since the development of clinical SLE requires IgG antibodies against DNA, it was predicted that no autoimmunity would develop in these mice [43]. The results were as expected. These findings supported the B-cell causation theory of SLE, and led me to the thesis that drugs that control B cells would be fundamental therapeutic agents for autoimmune diseases.

## 7 History of anti-human IL-6 receptor antibody

### 7.1 Polyclonal B cell activation

In 1969, after graduating from the Post Graduate School of Osaka University, School of Pharmaceutical science, I joined Chugai Pharmaceutical Co., Ltd. to work at a Central Research Laboratory responsible for new drug research and development. I was recommended to study immunology by the director of the Research Lab. Initially, I worked on searching for new immunosuppressants and anti-allergic agents, but in time, I became more interested in autoimmune diseases. I was motivated by the first-in-Japan anti-rheumatic drug, lobenzarit, developed by Chugai, and I was heavily involved in the research and development of this unique drug. Since lobenzarit has neither immunosuppressive nor anti-inflammatory effects, we did not know why it was effective in rheumatoid arthritis [44], but one day at the library I found a paper about an immunostimulatory agent named levamisole which appeared to activate suppressor T cells, resulting in inhibition of arthritis development.

To confirm this hypothesis, I wanted to investigate the mechanism of action for lobenzarit in the laboratory of the Division of Rheumatology, Allergy and Clinical Immunology at the University of California, Davis, School of Medicine. There I met M. Eric Gershwin who had just been moved from the NIH. He was one of the researchers arguing that a reduced function of suppressor T cells triggered autoantibody production. This is why I chose his lab. Between 1978 and 1981, I worked with Dr. Gershwin and was in charge of studies using NZB/NZWF1 mice that spontaneously develop autoimmune diseases. As described above, the conclusion of my work during my stay in Davis was that the phenomenon of polyclonal B cell activation was a primary defect in autoimmune disease-prone NZB/NZW F1 mice.

### 7.2 Search for B-cell inhibitors

Based on the results obtained during my studying abroad, I began to consider that the fundamental treatment of the autoimmune disease may be facilitated by discovery of a B cell inhibitor. However, it was not known what causes B cell activation. In 1982, I began to work with Dr. Takuya Katagiri of the University of Tokyo to identify causative factors. As a result, the activity which induces B cell to proliferate and differentiate into antibody producing cell was found in the soluble fraction obtained from homogenized lymph nodes of mice with autoimmune disease [45].

Soon after IL-6 was discovered as a B cell differentiation factor, a paper indicating that IL-6 may play an important role in autoimmune diseases was published [46]. Very interestingly, this finding was derived from clinical findings in cancer patients and not in patients with autoimmune diseases. A patient with cardiac myxoma had clinical symptoms including fever, general malaise and arthralgia. Laboratory tests demonstrated hyper-γ-globulinemia and positive autoantibody, high serum C-reactive protein and hypoalbuminemia, as well as increased erythrocyte sedimentation rate (ESR), all of which are typical chronic inflammatory markers. When the tumor was resected, all of these symptoms disappeared and when these tumor cells were cultured in vitro, IL-6 was released into the culture medium.

### 7.3 Industry-academia collaboration

In 1986, the same year that the IL-6 gene was discovered, a collaboration between Chugai Pharmaceutical and Osaka University began with the aim of developing IL-6 inhibitors. During the first meeting of the Chugai-Osaka Joint Research Team, we decided to focus on cloning a gene coding for the IL-6 receptor in order to clarify the signaling mechanism of IL-6. Chugai was planning to use the extra-cellular portion of the receptor as an IL-6 inhibitor. IL-6 concentrations were measured in the blood and urine of patients with rheumatoid arthritis, systemic lupus erythematosus and glomerulonephritis. In collaboration with Dr. Shiro Shimizu of Kanazawa medical Univ., higher levels of IL-6 were found in the blood and synovial fluid of patients with rheumatoid arthritis [47].

### 7.4 Continuum of difficulties and then luck

In 1988, the cDNA coding for the receptor was cloned by Kishimoto’s group. At Chugai, the extracellular regions of the receptors were cut at various positions (soluble receptors) and tested for their ability to bind IL-6. As a result, we found that the soluble receptor containing the 344th amino acid from the N-terminal had binding ability to IL-6. However, we were very much disappointed because of unanticipated events. Although it binds to IL-6, it could not inhibit the biological function of IL-6. Although it took nearly a year to explain the reason for this, we finally found that a second receptor (GP130) was needed, and the mechanism of transmission of the signal into the cells was clarified. As described above (Fig. 4), the soluble receptor act as an agonist and not as an antagonist. In addition to this, when the inhibitory activity was examined by a random screening of the small molecular weight compounds chemically synthesized, and by synthesizing the constituent peptide fragments of IL-6 and the IL-6 receptor, no effective inhibitors were found [48].

In 1990, we found that a mouse anti human IL-6 receptor monoclonal antibody was able to inhibit the binding of IL-6 to IL-6 receptors. We decided to make this a candidate for drug development. However, there was difficulty in administration of murine antibodies to humans due to its antigenicity. Luckily, genetic engineering technology called “CDR-grafting” had been established and published [49]. However, there was another big problem as the technology for commercially producing large quantities of the humanized antibody had yet to be established.

**Fig. 7:**
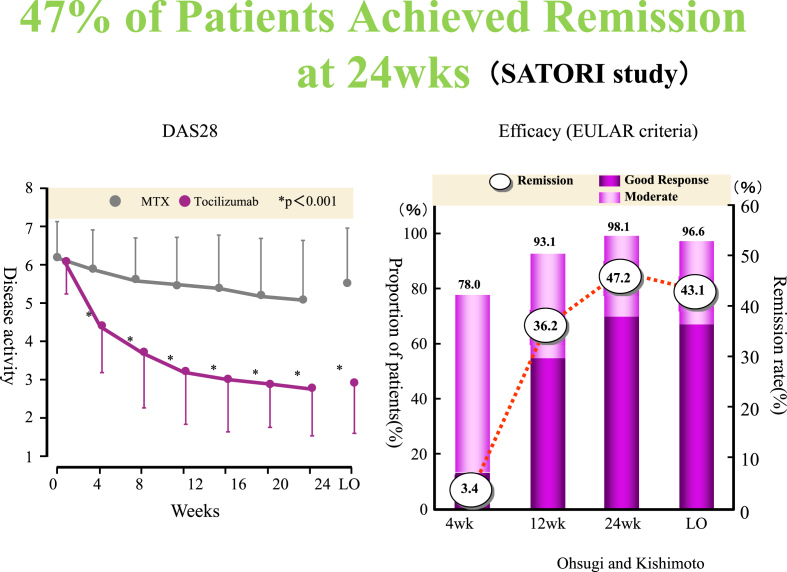
Clinical phase III study (named “Satori Study”). Approximately half of the patients had achieved clinical remission by the 6-month evaluation. (Left) The vertical axis is the DAS28 value (disease activity). Data is shown as an average value of all cases and standard error (because it gets complicated, the vertical axis showing the standard error shows only on one side). The horizontal axis shows the time (in weeks) from the time that tocilizumab was administered (1 time every 4 weeks). The DAS28 value is not fluctuating much in the control group, but in the group to which tocilizumab was administered, it is obviously decreasing, showing improved symptoms. In any of the 4-week administration periods, statistically significant improvement can be recognized (marked with a * symbol). (B) The vertical axis on the left side is the percentage (%) of patients that achieved improvement in their symptoms (complete response and effect). The vertical axis on the right side shows (bar graph) the DAS28 remission rate (see Note below). As administration continued, the ratio of patients with improved symptoms increased, and after 24 weeks (after 6 administrations), the ratio of patients who have achieved “Complete Response” or “Effect” reached 98%. The number inside the circle shows the DAS28 remission rate (dotted line polyline graph). After 24 weeks, 47% achieved remission. (Note) DAS28. Used as a standard for showing disease activity level using a score calculated based on a calculation method established by EULAR (the European League of Arthritis and Rheumatism). Sum total of the number of joints showing inflammation or pain amongst 28 fixed joints, and erythrocyte sedimentation rate, and for total symptoms, multiplying their respective values by an established coefficient. If the DAS28 score is below 2.6, it is considered remission. (Adapted from Nishimoto N, Miyasaka N, Yamamoto K, Kawai S, Takeuchi T, Azuma J, Kishimoto T. Study of active controlled tocilizumab monotherapy for rheumatoid arthritis patients with an inadequate response to methotrexate (SATORI): significant reduction in disease activity and serum vascular endothelial growth factor by IL-6 receptor inhibition therapy. Mod Rheumatol. 2009; 19:12–19).

Interestingly, Kawano of Hiroshima University reported that IL-6 acts as a growth factor for multiple myeloma cells [50]. Based on this finding, Chugai decided to develop a drug for multiple myeloma. Since the MRC (Medical Research Council) Collaborative Center in the United Kingdom held the intellectual property rights for antibody humanization technologies, we decided to collaborate with them, and a humanized anti IL-6 receptor antibody was completed in 1991 [51]. This was later named tocilizumab (Fig. 1). Because of a price gap between manufacturing costs and drug prices, the development of IL-6 mAb for autoimmune diseases was initially abandoned, and the focus was diverted toward the development of drugs for multiple myeloma instead. However, in 1995, a chimeric antibody against TNFα (infliximab) was found to have a remarkable therapeutic effect on rheumatoid arthritis. Infliximab was introduced to Tanabe Pharmaceutical, a Japanese pharmaceuticals by Centcore Co., Ltd., a start-up company in the United States. It was known that TNFα had the ability to induce IL-6 gene expression. Therefore, it was easily imagined that at least part of the pharmacological effects of infliximab were exerted through inhibiting the production of IL-6. This expectation was proven correct, and tocilizumab was found to markedly improve symptoms in patients with rheumatoid arthritis [1,2] (see Fig. 7).

### 7.5 Impact of tocilizumab development on medicine and pharmacy

Although it has been well known that high levels of IL-6 were detected in the joint fluid of rheumatoid arthritis patients and correlated with disease activity [52,53], the question whether this abnormality was a cause or result of disease remained a mystery. Until the advent of tocilizumab, TNFα was thought to be the main cause of the rheumatoid arthritis. The prominent symptomatic response to tocilizumab has led to the gradual recognition that IL-6 plays a crucial etiologic role. This was supported by subsequent Japanese and foreign phase III studies. Tocilizumab has a great impact on the effectiveness of treatment even in cases where a satisfactory therapeutic effect is not obtained with TNFα inhibitors, and thus it was called a rescuer for patients who had lost all their treatment options [1,2].

Tocilizumab is the only biologic that demonstrated superiority in clinical efficacy in direct comparison trials with the standard therapy methotrexate. Therefore, tocilizumab monotherapy is recommended in cases where methotrexate cannot be used due to adverse reactions, etc. (updated as EULAR Recommendations 2016 UPDATE, a guideline for the treatment of rheumatoid arthritis, at the European Society of Rheumatology, EULAR, June 2016). The study of tocilizumab has led to new findings, such as IL-6 playing major roles in angiogenesis and cartilage-bone destruction, which are important for the progression of rheumatoid arthritis.

Very importantly, a recent clinical study (The REBONE study) demonstrated that tocilizumab monotherapy achieves more pronounced repair of existing bone erosions than ADA/MTX combination therapy. Hence, IL-6 is a central factor for the disturbed bone homeostasis in the joints of patients with RA [54].

IL-6 is also closely involved as a central causative agent in the development of anaemia associated with chronic inflammation. It has also been implicated in general malaise, decreased appetite, fever and pain with marked improvement observed with tocilizumab treatment. In addition, it has also been shown that IL-6 receptor antibodies exert their effects through the inhibition of differentiation of Th17 ​cells in various animal models [[55], [56], [57]]. Recently, studies have reported that the number of regulatory T cells increases in patients who respond to tocilizumab treatment [58].

## 8 Milestones in the development of tocilizumab

I would like to mention four critical avenues to the success of the drug discovery:

The first was a period of searching for a B cell inhibitor without any appropriate assay methods, which was like walking in the dark. To overcome this, we had to clarify what is the causative factor for the polyclonal B cell activation phenomenon, which we believed was the main cause of autoimmune diseases. This crisis was averted by the discovery of IL-6 by Kishimoto and his colleagues.

The second crisis occurred during a period when no candidates for inhibiting IL-6 were found. This crucial moment was rescued by the development of antibody engineering technology, which was the state-of-the-art technique of humanizing mouse antibodies.

Thirdly, Kawano discovered that “IL-6 is a growth factor for multiple myeloma cells.” In the absence of this discovery, the research project for tocilizumab could not be continued as it couldn’t overcome the barriers of the gap between production costs and drug price.

Finally, the fourth critical path was the advent of infliximab in the US and its export to Japan. This awakened experts who were involved in the drug discovery field in the pharmaceutical companies in Japan, who thought that antibodies would never be a therapeutic option for the treatment of patients with autoimmune diseases.

## Conflict of interest

None.
